# Multifocal Extranodal Involvement of Diffuse Large B-Cell Lymphoma

**DOI:** 10.1155/2013/794642

**Published:** 2013-09-15

**Authors:** Devrim Cabuk, Y. Taha Gullu, Ilknur Basyigit, Ozgur Acikgoz, Kazım Uygun, Kursat Yildiz, Fusun Yildiz

**Affiliations:** ^1^Department of Medical Oncology, Kocaeli University Hospital, Umuttepe, 41380 Kocaeli, Turkey; ^2^Department of Pulmonary Disease, Kocaeli University Hospital, Umuttepe, 41380 Kocaeli, Turkey; ^3^Department of Pathology, Kocaeli University Hospital, Umuttepe, 41380 Kocaeli, Turkey

## Abstract

Endobronchial involvement of extrapulmonary malignant tumors is uncommon and mostly associated with breast, kidney, colon, and rectum carcinomas. A 68-year-old male with a prior diagnosis of colon non-Hodgkin lymphoma (NHL) was admitted to the hospital with a complaint of cough, sputum, and dyspnea. The chest radiograph showed right hilar enlargement and opacity at the right middle zone suggestive of a mass lesion. Computed tomography of thorax revealed a right-sided mass lesion extending to thoracic wall with the destruction of the third and the fourth ribs and a right hilar mass lesion. Fiberoptic bronchoscopy was performed in order to evaluate endobronchial involvement and showed stenosis with mucosal tumor infiltration in right upper lobe bronchus. The pathological examination of bronchoscopic biopsy specimen reported diffuse large B-cell lymphoma and the patient was accepted as the endobronchial recurrence of sigmoid colon NHL. The patient is still under treatment of R-ICE (rituximab-ifosfamide-carboplatin-etoposide) chemotherapy and partial regression of pulmonary lesions was noted after 3 courses of treatment.

## 1. Introduction 

Endobronchial involvement of extrapulmonary malignant tumors is uncommon and mostly associated with breast, kidney, colon, and rectum carcinomas [1, 2]. Although the lung is a frequent site for lymphoma involvement, endobronchial metastasis of non-Hodgkin lymphoma (NHL) is extremely rare.

Extranodal lymphomas originating in solid organs account for one-third of all cases of NHL. Gastrointestinal (GI) tract is the most common site of extranodal lymphomas. GI tract lymphomas occur most commonly in the stomach and colorectal NHL accounts for only 10–20% of them [3].

NHL originates from B or T lymphocytes. Mucosa-associated lymphoid tissue (MALT) and diffuse large B-cell lymphoma (DLBCL) are the most commonly observed histological subtypes in the GI tract [4]. DLBCL of the GI is an aggressive lymphoma which more commonly affects males with a median age of 50–60 years [5]. The reported 5-year survival is relatively poor, ranging between 27 and 55%.

Here, we present a patient with endobronchial involvement of diffuse large B-cell lymphoma who has completely remitted sigmoid colon NHL as a primary site.

## 2. Case Report

A 68-year-old male was admitted to the hospital with a complaint of diarrhea, abdominal pain, weight loss, and hematochezia. Ulcerated plaque-like lesions with local necrotizing areas along the 10 cm segment of sigmoid colon were detected in colonoscopy. The pathological examination of biopsy material was suggestive of malignancy and left hemicolectomy was performed. The patient was diagnosed as stage II diffuse large B-cell lymphoma based on surgical biopsy and radiological findings. The patient was treated with 8 courses of R-CHOP (rituximab, cyclophosphamide, doxorubicin, vincristine, and prednisolone) chemotherapy followed by pelvic-paraaortic radiotherapy (RT). Complete remission was achieved with the treatment with no signs of recurrence in the following radiological examinations.

**Figure 1:**
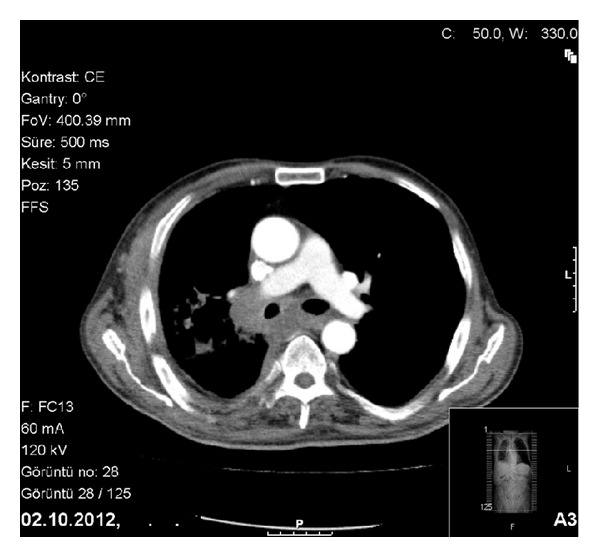
Subcarinal and right hilar lymphadenopathies in thorax CT.

**Figure 2:**
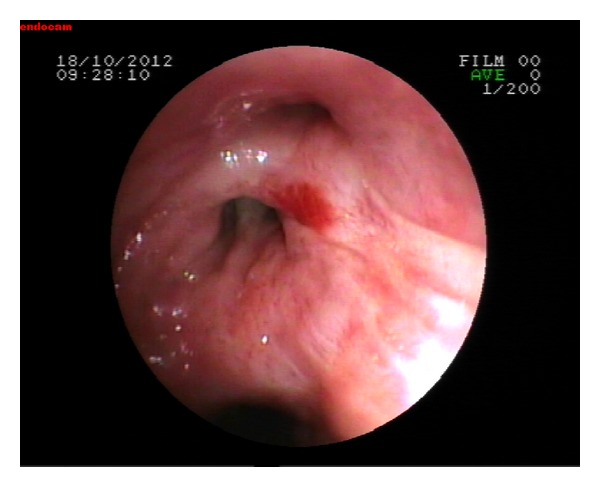
Stenosis with mucosal tumor infiltration in right upper lobe bronchus.

**Figure 3:**
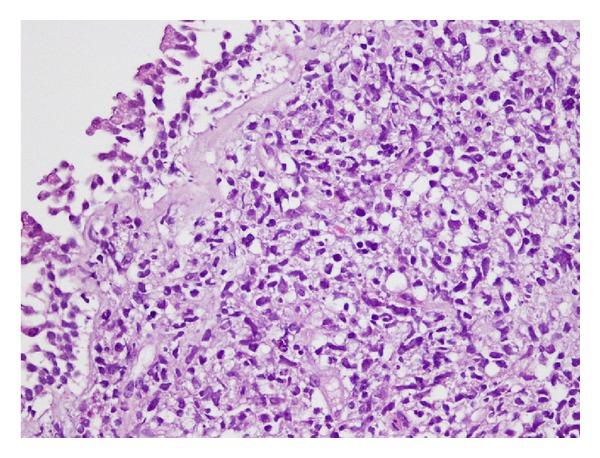
The figure demonstrates section belonging to bronchial mucosa. There is diffuse infiltration of atypical lymphoid cells under regular bronchial epithelium.

Patient was readmitted with the symptoms of cough, sputum, and dyspnea three months after the treatment completion. He was good in clinical condition, his heart rate was 92/min, respiratory rate was 26/min, blood pressure was 130/80 mm Hg, and temperature was 38.2°C on physical examination. Chest examination indicated decreased breath sounds over the middle zone of right hemithorax. The chest radiograph showed right hilar enlargement and opacity at the right middle zone suggestive of a mass lesion. Computed tomography of thorax revealed a right-sided mass lesion extending to thoracic wall with the destruction of the third and the fourth ribs and a right hilar mass lesion obstructing the right upper lobe and intermediate bronchus with a postobstructive consolidation. There were subcarinal and right hilar lymphadenopathies accompanied with parenchymal findings (Figure 1). Fiberoptic bronchoscopy (FOB) was performed in order to evaluate endobronchial involvement and stenosis with mucosal tumor infiltration in right upper lobe bronchus was detected (Figure 2). The pathological examination of bronchoscopic biopsy specimen was reported as diffuse large B-cell lymphoma and the patient was accepted as the endobronchial recurrence of sigmoid colon NHL (Figure 3). The patient is still under treatment of R-ICE (rituximab-ifosfamide-carboplatin-etoposide) chemotherapy and partial regression of pulmonary lesions was noted after 3 courses of treatment.

## 3. Discussion

We presented an endobronchial diffuse large B-cell lymphoma in a patient with colon DLBCL in which complete remission was achieved and no recurrence was noted during three months of followup. 

DLBCL is an aggressive form of non-Hodgkin lymphoma and comprises approximately 30% of all lymphomas. It usually occurs in lymph nodes while extranodal presentation most commonly involves the gastrointestinal tract, bone marrow, and skin [6]. 

Primary pulmonary lymphoma usually presents as MALT lymphoma while lung DLBCL is reported only in case reports. The most frequently reported pulmonary involvement is a lung mass usually greater than 5 cm in diameter [7]. Pleural involvement presents as an important extranodal site for DLBCL, which is also found to be associated with overall survival [8]. 

In a series consisting of 82 patients with median age of 61 years, Neri et al. found that the most frequent symptoms were cough and chest pain and no B symptoms were present in primary lung DLBCL patients. They have reported complete remission in 94% of the patients, reported overall survival rate of 92%, and suggested that primary lung DLBCL is a disease with good prognosis [7].

On the other hand, extranodal involvement is considered as a poor prognostic factor in patients with DLBCL. Takahashi et al. reported a retrospective study evaluating the prognostic impact of each extranodal site in 1221 patients with extranodal DLBCL. Lung involvement was found in 3.7% of the patients and additional pleural involvement was also reported in 5.2% of them [8]. Although characteristics of lung involvement are not mentioned in this paper, previous reports suggested that endobronchial lesions are extremely rare in DLBLC and they are generally associated with enlarged mediastinal lymph nodes and bilateral infiltration of lung parenchyma and/or mass lesion [9]. Endobronchial lymphoma is classified into two types, according to pattern of involvement. Type I includes diffuse submucosal infiltrates originating from hematogenous or lymphangitic spread in the presence of systemic lymphoma. Type II (similar to our patient) includes airway involvement by a localized mass due to direct spread of lymphoma from adjacent lymph nodes or arising de novo from bronchus-associated lymphoid tissue (BALT). Type II lesions have been associated in all instances with signs of airway obstruction such as cough or wheezes [10]. Furthermore, the majority of the cases with extranodal DLBCL were presented as solitary extranodal site and multiple extranodal involvements were reported in about 20% of the cases. Recent case report from Sakai et al. suggested that multiple extranodal DLBCL might be associated with chromosome translocations [11].

In conclusion, endobronchial DLBCL accompanied by colon lymphoma is extremely rare in previous reports. Localized endobronchial lesion resembling primary lung carcinoma might be observed in lymphoma especially in cases with underlying extranodal NHL and bronchoscopic evaluation is essential in order to make the differential diagnosis.
